# The Role of Ketogenic Diet in Treatment of Mental Illnesses

**DOI:** 10.1192/j.eurpsy.2025.2011

**Published:** 2025-08-26

**Authors:** A. V. Samokhvalov

**Affiliations:** 1 Dr. Sam’s Health; 2 ASU/CPC/Interventional Psychiatry, Homewood Health Centre, Guelph, Ontario; 3 Department of Psychiatry & Behavioural Neurosciences, McMaster University, Hamilton, Ontario; 4 Homewood Research Institute, Guelph, Ontario, Canada

## Abstract

**Introduction:**

Ketogenic Diet (KD) has been introduced as a treatment option for epilepsy over a century ago and since then it had gained and lost its popularity. Its current revival is heavily sensationalized and KD is promoted as a cure-it-all for a wide range of mental illnesses. The present review aims to review the validity of the evidence supporting KD use in psychiatry.

**Objectives:**

1. To review the origins and types of ketogenic diet as well as the possible rationale for its use in psychiatry
2. To provide a narrative review of effectiveness of ketogenic diet for management of major mental illnesses

**Methods:**

Scoping review of research articles published in PubMed without restrictions in terms of language or publication date.

**Results:**

We conducted a scoping review using a wide range of keywords. There are multiple studies dedicated fully or partially on ketogenic diet and a variety of mental illnesses. Majority of studies are focused on low-carbohydrate diets and not necessarily on KD per se, which complicates interpretation of findings. At the same time, we were able to identify multiple case studies and case series as well as some open-label and placebo-controlled studies involving small groups of participants receiving KD mostly as an adjunct treatment for their respective condition. There was a significant degree of heterogeneity both in terms of methodological aspects and clinical outcomes of these studies, which do not allow for further data synthesis at this point. At the same time, there are some indications that KD may be useful in certain clinical scenarios.

**Conclusions:**

The objective data on impact of ketogenic diet on major mental illnesses are scarce, but show some promise. Further research is needed to elaborate both the mechanisms and the degree of impact of ketogenic diet on mental health. There is not enough evidence to suggest that ketogenic diet is an independent treatment option for any psychiatric condition, but at the same time it can be suggested as an adjunct measure.

**Disclosure of Interest:**

None Declared

